# Incidental Gallbladder Carcinoma in Gallbladder Polyps: Challenges of Gallbladder Malignancy for an Endemic Population

**DOI:** 10.21315/mjms2021.28.1.4

**Published:** 2021-02-24

**Authors:** Abhishek Gautam, Anshuman Pandey, Shakeel Masood, Smita Chauhan, Dinesh Choudhary, Suneed Kumar, Shibumon Madhawan, Sneha Jha, Vijay Saini

**Affiliations:** Department of Surgical Gastroenterology, Dr Ram Manohar Lohia Institute of Medical Sciences, Lucknow, Uttar Pradesh, India

**Keywords:** gallbladder polyp, gallbladder carcinoma, endemic area, size

## Abstract

**Background:**

Gallbladder polyps are considered pre-malignant lesions of gallbladder carcinoma. This study aims to highlight the role of early cholecystectomy in the management of gallbladder polyps in an endemic population.

**Methods:**

A retrospective analysis of 2,076 lap cholecystectomy procedures performed at the Department of Surgical Gastroenterology at a tertiary referral centre in Northern India was conducted and incidental malignancy in gallbladder polyps analysed. The 8th edition of the American Joint Committee on Cancer for tumour-node-metastasis (TNM) staging of gallbladder carcinoma was used.

**Results:**

Of 54 patients with gallbladder polyps, 53 had benign histology and one had malignant cells in the lamina propria suggestive of T1a adenocarcinoma. The patient with the malignant polyp was older (57 years old) than the patients in the non-cancer group, which had a mean age of 45 (*P* = 0.039). The size of the malignant polyp was approximately 4 mm, significantly smaller than the average 7.9 mm size of the benign polys (*P* = 0.031).

**Conclusion:**

Cholecystectomy needs to be considered early in the management of small-sized gallbladder polyps, particularly in areas endemic for gallbladder carcinoma.

## Introduction

Gallbladder polyps are elevations of the gallbladder wall that protrude into the lumen. They are frequently detected on abdominal ultrasound and have a prevalence of 3%–7% in the global adult population (1). Gallbladder polyps have been associated with the risk of malignancy, which ranges from 0%–27% depending upon the size and morphology of the polyp and the age of the patient. In approximately 4%–9% of cases, gallbladder polyps are associated with gallstones (2).

Gallbladder carcinoma is a highly aggressive malignancy with a generally poor prognosis. The incidence varies significantly between ethnic groups. The Gangetic belt region in Northern India has one of the highest incidence rates, ranging from 10–22 in every 100,000 people. In addition, the average age of presentation in India is one decade earlier than in Western countries. As the prognosis of gallbladder carcinoma worsens in later stages of the disease, it is imperative to detect and manage it early. Because gallbladder polyps are a risk factor for gallbladder carcinoma, early diagnosis and appropriate management are of paramount importance. However, it is a diagnostic challenge to determine which polyps are likely to be malignant or undergo malignant transformation. Many asymptomatic patients may harbour malignancy and to follow up these patients in a rural scenario is extremely challenging. However, early detection of malignant gallbladder polyps may potentially be curative because most lesions are limited to the mucosa. The existing literature suggests the most consistent predictors of malignant gallbladder polyps are older age (> 50 years old), size (> 10 mm) and having a single polyp. Based on available data, current practice recommendations are to perform cholecystectomy for gallbladder polyps larger than 10 mm. These recommendations may not hold in an endemic population, as younger patients with smaller polyps may harbour malignancy. Cholecystectomy is one of the most commonly performed surgical procedures worldwide and has very low morbidity. Performing a cholecystectomy on patients with gallbladder carcinoma limited to the mucosa offers the chance of a cure for a disease that otherwise has a poor prognosis.

The aim of our study is to emphasise that even a small asymptomatic gallbladder polyp may harbour malignancy and that not offering a potentially curative simple cholecystectomy in such cases may constitute mismanagement. The present study was conducted to analyse gallbladder polyps in a population in which gallbladder carcinoma is endemic. Even small polyps (4 mm) may harbour malignancy, and a simple cholecystectomy at an appropriate time offers the chance of cure for a disease associated with a generally poor survival rate and that may later progress rapidly and become inoperable.

## Methods

A retrospective study was conducted at the Department of Surgical Gastroenterology of the Dr Ram Manohar Lohia Institute of Medical Sciences (RMLIMS) in Lucknow, India between January 2011 and December 2019. It included 54 patients with polypoid lesions of the gallbladder who underwent laparoscopic cholecystectomy. A total of 2,076 laparoscopic cholecystectomy procedures were performed during this period, with the most common indication being symptomatic cholelithiasis. Patients with a gallbladder polyp and gallstones (*n* = 7) were excluded. Patients’ demographic data and clinical symptoms and signs were recorded, and laboratory investigations, pre-operative ultrasonography of the abdomen, and computed tomography of the abdomen were performed for all patients presenting with gallbladder polyps. The following standardised United States criteria were used to identify polyps: immobile, hyperechoic compared to the surrounding bile, non-shadowing and attached to the gallbladder wall. After a complete pre-operative work-up, a fitness test for laparoscopic cholecystectomy, and giving informed consent, patients underwent surgery. Respective representative specimens were sent for routine histopathologic examination. The primary objective was to consider further refinement of the size criteria in the Indian population in order to consider gallbladder polyp patients for surgery. The secondary objective was to emphasise the need for consensus guidelines to be followed for the surveillance of gallbladder polyp patients in the endemic population.

All symptomatic patients and all asymptomatic patients with a polyp larger than 10 mm who underwent cholecystectomy were included in the study. All gallbladder polyp patients with i) gallstone disease or evidence of sludge and related complications; ii) porcelain gallbladder or iii) focal or diffuse thickening of the gallbladder wall were excluded. The hospital data was analysed retrospectively, and any association between a small polyp and age and malignancy was assessed. Approval was given by the ethics committee of the institute. There was no funding involved in the study and no conflicts of interest. The personal data of all patients was secured in the hospital’s native data system. The data were analysed with IBM SPSS Statistic version 12.0 for Windows (IBM, Armonk, NY). Baseline demographics were assessed using mean ± SD or median for numerical data and percentages for categorical data. A *P*-value of < 0.05 was considered statistically significant.

## Results

Of the 54 patients who underwent surgery, the median age was 44 years old (range 36–64 years old) and the median size of polyps on ultrasound was 7.6 mm (range 3 mm–14 mm). Twenty-five (54%) patients were male and 29 (46%) were female. The majority (43 patients, 79.6%) had pain in the right hypochondrium, indicating that symptom criteria may be the most important factor in making a decision regarding surgery in a clinical setting (Table 1). Other patients complained of vague abdominal pain, dyspepsia, fatigue and loss of appetite.

A histopathologic finding of benign polyps was noted in 53 patients (98.14%), while one patient (1.86%) had a malignant polyp (T1a adenocarcinoma). Gender, number of polyps, body mass index, total bilirubin, direct bilirubin, aspartate aminotransferase (AST), alanine aminotransferase (ALT), alkaline phosphatase (ALP), fasting glucose, thyroid profile and carcinoembryonic antigen (CA) 19-9 were not significantly different between the patients who had benign polyps and the patient with malignancy (Table 2).

**Figure 1:**
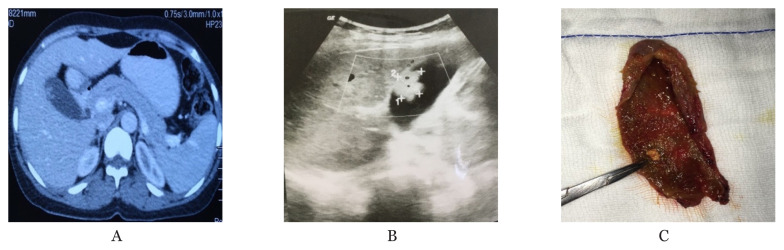
**A**. Portal venous phase CECT abdomen of a 49-year-old male who underwent laparoscopic cholecystectomy for GB polyp presenting with right hypochondrial pain. Final HPE turned out to be benign. **B**. Transabdominal ultrasound imaging in supine position of the same patient, as in the first figure. An intraluminal hyperechoic lesion with no acoustic shadowing with the typical appearance of ‘ball on the wall’ appearance is seen on the hepatic surface of the gallbladder. **C**. Intraoperative image of a gallbladder after cut open. On macroscopic examination, nearly 5 mm polyp can be seen close to the neck of the gallbladder. It is imperative to examine the whole specimen cut open carefully, not be missed any focal thickening or presence of any nodule

**Figure 2:**
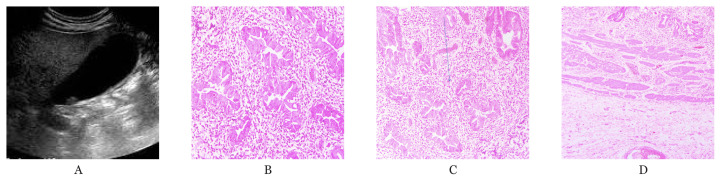
**A**. Transabdominal ultrasound of 57 years old patient with malignant polyp. Polyp is appreciated on the peritoneal side of gall bladder with no evidence of any focal thickening or stone. **B**. Microscopic image of tumour cells deposited in the lamina propria of the gallbladder wall. **C**. Well differentiated adenocarcinoma within the lamina propria. **D**. Muscle layer is free of the tumour cells (H&E original magnification 40×)

The patient with the malignant polyp was older (57 years old) than the patients in the non-cancer group, the mean age of which was 45 years old (*P-*value = 0.039). The size of the malignant polyp was 4 mm, significantly smaller than the benign polyps with an average size of 7.9 mm (*P*-value = 0.031) (Figure 1). The patient with the malignant polyp complained of pain in the right hypochondrium with no loss of appetite or weight loss. Ultrasonography revealed a single polyp of 4 mm on the peritoneal side of the gallbladder with no associated sludge or stone or wall thickening (Table 3). Histopathology showed tumour cells deposited within the glands in the lamina propria (Figure 2). The patient was diabetic, which was well controlled by medications. Of the patients with benign polyps, eight had diabetes and six had hypertension. Eleven patients in the benign group were asymptomatic and the indication for cholecystectomy for these patients was a polyp larger than 10 mm. Depending on the available data and considering the sample population in our study, a Fisher’s exact test was used to calculate the statistical significance of various parameters at a significant *P*-value of 0.05 and power of 80%. Two characteristics — older age (*P =* 0.039) and small polyp size (*P =* 0.031) — independently showed a positive association with malignancy of the gallbladder polyp.

## Discussion

**Figure 3:**
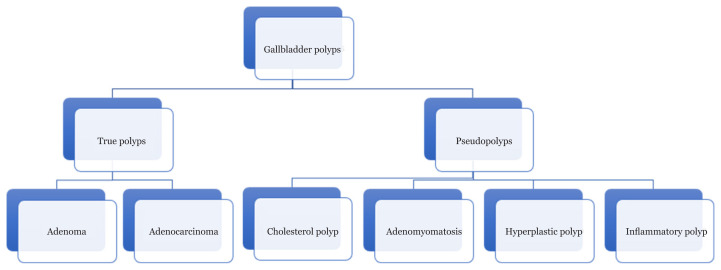
Classification of gallbladder polyps

Gallbladder polyps are protrusions of the gallbladder wall into the lumen. Morphologically, they can be sessile or pedunculated. They are common lesions that should not be ignored because of their association with malignancy. Previous studies have reported an incidence of gallbladder polyps of 0.3%–12.0% upon routine health examinations of the general population (4, 5). Generally, these lesions are categorised as true polyps or pseudo polyps (Figure 3). Only true polyps are known to have malignant potential. Pseudo polyps are fairly benign lesions, with cholesterol polyps being the most common gallbladder polyp.

Multiple risk factors associated with gallbladder polyps have been linked with malignancy. Most significant of these are age and polyp size (11, 15). Age over 50 years old and polyp size over 10 mm predispose one to gallbladder malignancy. Regarding polyps of less than 10 mm, the maximum risk is found in patients over 50 years old with a single sessile polyp (11, 16) (Table 4). Elevated carcinoembryonic antigen or CA19-9 have not been found to correlate with malignancy (14).

The deciding factor that determines the management of gallbladder polyps is polyp size. There is no established consensus regarding the exact size of an asymptomatic polyp to be considered for surgery; some studies consider it to be 10 mm and others consider it to be 12 mm (11, 15). There is a greater risk of malignancy in polyps larger than 10 mm, but the literature also highlights malignancy in polyps smaller than 10 mm. The data is sparse, particularly in populations where gallbladder carcinoma is endemic. A prospective study of 1204 patients with gallbladder polyps for 2 years found an increased risk of malignancy in polyps larger than 3 mm and recommended cholecystectomy as a diagnostic tool to rule out malignancy (18).

There exists controversy and lack of consensus regarding the frequency and duration of follow-up of patients with polyps smaller than 10 mm. A systematic review recommended following-up on polyps that are 5 mm–10 mm using ultrasound at 6 months and 12 months, and subsequent individually tailored surveillance plans (16). The joint guidelines proposed in 2017 regarding the management of gallbladder polyps recommends ultrasound at 6 months and 12 months for asymptomatic, low-risk polyps of 6 mm–9 mm followed by yearly check-ups up to 5 years (15). A similar follow-up is recommended for asymptomatic polyps smaller than 6 mm in patients with risk factors for malignancy (age over 50 years, sessile polyp, primary sclerosing cholangitis and Indian ethnicity), whereas imaging is recommended at 1, 3 and 5 years for low-risk, asymptomatic polyps smaller than 6 mm. The group recommends cholecystectomy in cases where polyps are 6 mm–9 mm with a high risk of malignancy. Indian ethnicity is considered a higher-than-normal risk for malignancy and cholecystectomy is recommended for all patients of Indian ethnicity with polyps larger than 6 mm. A similar recommendation was made by the Society of Endoscopic and Laparoscopic Surgeons of India, particularly for those over 50 years old (17).

Follow-up imaging may have a limited benefit, as only a small number of polyps actually change in size during the follow-up period. A systematic review identified 10 studies that looked at the follow-up of gallbladder polyps between 6 months and 7 years (17). It found that only 7.6% of polyps increased in size. Another study found that that 93% of polyps did not change in size during the follow-up period (17). Neither study stated if growth was more likely to be seen in pseudo polyps or true polyps.

A significant issue regarding the follow-up of smaller polyps in the Indian scenario is the loss to follow-up. Most patients reside in rural areas and do not have immediate access to proper imaging at peripheral centres and regular follow-up is not prioritised. The individual user-dependent nature of ultrasound as an imaging modality may also be a concern in routine follow-up at peripheral centres. Living in a rural area is itself associated with an increased risk of malignancy. A prospective study found that 80% of patients with gallbladder carcinoma resided in rural areas compared to 54% of patients with gallstones (OR 3.52; 95% CI: 2.48–4.99) (19). In such a scenario, cholecystectomy should be an option, particularly for an endemic population. Most studies highlighting the significance of polyp size as a criterion for gallbladder polyp surgery have been conducted in populations with a low incidence of gallbladder malignancy.

Gallbladder cancer is an aggressive malignancy that has an extremely poor prognosis. The only chance of cure is early detection and curative surgery (4). There is significant variation in the incidence rates of gallbladder cancer based on geographic and ethnic grounds. The North Indian Gangetic belt is considered to have about 10% of the global gallbladder carcinoma burden, and the incidence of gallbladder carcinoma in India is rising steadily (23). According to population-based cancer registries set up by the Indian Council of Medical Research, the average age-adjusted rate among women in India increased from 6.2/100,000 in 2001–2004 to 10.4/100,000 in 2012–2014 (20, 21). It is difficult to detect tumours early on because the gallbladder mucosa is not amenable to direct endoscopic inspection. The 5-year survival rate is often less than 5% (22), whereas the 5-year survival in cases of tumours limited to the mucosa is nearly 100%. This indicates that if detected early, gallbladder disease can be virtually cured by cholecystectomy, as in the case of small gallbladder polyps.

The present study assumed that malignancy in a gallbladder polyp is closely related to size. However, one 57-year-old patient had incidentally detected malignancy within a symptomatic 4 mm polyp (*P* = 0.031). The histopathologic staging was T1a and therefore index surgery was considered to be curative. In the case of the absence of symptoms in this patient, current management would have actually resulted in missing a cancerous lesion and later presentation with inoperable or metastatic disease, which is usually the case with gallbladder cancer. Simple cholecystectomy provided a cure for this patient. Considering the North Indian population has one of the highest incidence of gallbladder carcinoma in the world and the generally poor prognosis of the disease, the best chance of a cure is early diagnosis and surgery. It is suggested that polyp size alone to determine a treatment plan for gallbladder polyps may be insufficient. The conventional threshold of 10 mm in size as a cut off for cholecystectomy may deny the appropriate treatment of patients with smaller polyps, particularly in an endemic population.

Our study adds to the growing literature on smaller polyps. Larger multi-centric, comprehensive studies are required in endemic areas to formulate recommendations. Currently, the scenario is individualised, as gallbladder polyps are quite a frequent finding upon routine abdominal ultrasound. Routine cholecystectomy seems inappropriate and will add to the health care burden; however, there needs to be a vigilant approach to managing gallbladder polyps in endemic areas, irrespective of polyp size. There is no conflict of interest in the present study.

## Conclusion

Gallbladder polyps are common. However, management must be vigilant, particularly in endemic areas. Patients with polyps larger than 10 mm should undergo a cholecystectomy, followed by histopathologic examination. A safe procedure with minimal complications, cholecystectomy should be considered for asymptomatic patients of rural background and for young patients with smaller polyps, as they may still harbour malignancy. The present article highlights the need to consider cholecystectomy for smaller polyps (< 10 mm) in areas where gallbladder carcinoma is endemic.

## Figures and Tables

**Table 1:** Symptoms of patients in the study population

Symptom	Benign group (*n* = 53)	Malignant patient (*n* = 1)
Asymptomatic	11 (20.7%)	0
Pain	42 (79.2%)	1
Anorexia	2 (3.7%)	0

**Table 2:** Baseline characteristics of patients in the study

Characteristics	Patients in benign group (*n* = 53)	Malignant patient (*n* = 1)	*P-*value
Age (in years)	44 (9)	57	0.039#
Male: Female	4:5	Male	
BMI (kg/m^2^)	24.7(6)	28.4	0.526
Diabetes	8	1	0.121
Hypertension	6	NA	
Hb (gm/dL)	10.4 (2.3)	11.6	0.483
White blood cells (cells/mm^3^)	8,340 (2,100)	9,400	0.394
Serum albumin (gm/dL)	3.2 (0.7)	3.8	0.292
ALT (U/L)	33 (24)	37	0.584
Total bilirubin (mg/dL)	0.92 (0.23)	0.71	0.476
GGT (U/L)	26 (20)	32	0.328
CA19-9 (u/mL)	3.2 (2)	2.09	0.162

Notes:

# statistically significant;

GGT = gamma-glutamyl transferase; Hb = haemoglobin; Fisher’s exact test has been used comparative; BMI = body mass index

**Table 3:** Imaging characteristics of patients in the study

Characteristics	Benign polyp (*n* = 53)	Malignant polyp (*n* = 1)	*P*-value
Average ultrasound size (mm)	8.2+/−4.9	4	0.031
Average number of polyps (mm)	1.2+/−1	1	0.894

Note: Size was found to be statistically significant using Fisher’s exact test

**Table 4:** Risk assessment for gallbladder malignancy when polyp size is less than 10 mm^16^

Risk factors	Odds ratio	Odds of malignancy	Probability of malignancy (%)
Single polyp	2.05	0.045	4.3%
Sessile polyp	7.32	0.161	13.9%
Patient age > 50 years old	11.83	0.260	20.7%
Sessile, single polyp	15.01	0.330	24.8%
Patient age > 50 years old, single polyp	24.25	0.534	34.8%
Patient age > 50 years old, sessile polyp	86.60	1.905	65.6%
Patient age > 50 years old, sessile, single polyp	177.52	3.905	79.6%
